# Individual Genetic Contributions to Genital Shape Variation between *Drosophila simulans* and *D. mauritiana*

**DOI:** 10.1155/2014/808247

**Published:** 2014-09-08

**Authors:** Hélène LeVasseur-Viens, Amanda J. Moehring

**Affiliations:** Department of Biology, Western University, BGS 2080, 1151 Richmond Street, London, ON, Canada N6A 5B7

## Abstract

External genitalia are one of the most rapidly evolving morphological features in insects. In the *Drosophila melanogaster* species subgroup, males possess a nonfertilizing external genital structure, called the posterior lobe, which is highly divergent among even closely related species. A previous study on this subgroup mapped two genomic regions that affect lobe size and four that affect lobe shape differences between *D. mauritiana* and *D. sechellia*; none of the regions affected both size and shape. Here, we investigate whether three of these significant regions also affect lobe size and shape differences between the overlapping species pair *D. mauritiana* and *D. simulans*. We found that the same three regions of *D. mauritiana*, previously shown to affect lobe morphology in a *D. sechellia* genetic background, also affect lobe morphology in a *D. simulans* genetic background, with one of the regions affecting both size and shape. Two of the regions also affected morphology when introgressed in the reciprocal direction. The overlap of regions affecting genital morphology within related species pairs indicates either that there is a common underlying genetic basis for variation in genital morphology within this species group or that there are multiple adjacent loci with the potential to influence genital morphology.

## 1. Introduction

Animal groups ranging from primates [1] to lizards [2] show rapid evolution of male genitalia. In addition to the inseminating, or primary, organs, external secondary organs involved in stimulation and copulation also exhibit rapid divergence in a variety of animal groups [3]. In insects, the divergence of male genitalia is so pronounced that even recently diverged sibling species show a high degree of variation in the male genitalia and/or secondary organs [4–6]. Several different models have been developed to explain the evolution of genitalia in individual species, but none explains why it occurs across so many animal groups [7, 8]. The most prominent competing theories that attempt to explain the pervasive occurrence of rapidly diverging male genitalia are the pleiotropy hypothesis, the lock and key hypothesis, and the sexual selection hypothesis [3, 7, 9–14]. While there is evidence supporting each of these models, sexual selection is thought to be the most prevalent influence on the divergence of male genitalia [3, 15, 16].

An understanding of the genetic underpinnings of genital shape enhances our ability to assess the evolutionary forces influencing genital morphology. One of the most widely used model systems for understanding the genetic basis of genital morphology is the* Drosophila melanogaster* species subgroup. These species are largely morphologically indistinguishable from one another except for the shape of the male's exterior genital lobes [17]. The bilaterally symmetrical posterior lobes, also called the genital arch, are a cuticular projection that surrounds the inverted adaegus. The lobes are inserted between the female's eighth and ninth abdominal tergites during copulation [18] and appear to be involved in several aspects of copulation and fertilization [19–21], making it likely that they experience sexual selection.

Several genetic mapping studies for lobe shape have been performed in this group, and while the maps identify genomic regions and not individual genetic loci, comparisons among studies can significantly enhance our understanding of how these sexually selected traits evolve. Most genetic mapping studies on genital morphology have used quantitative trait locus (QTL) mapping, but a recent study on the sibling species* D. mauritiana* and* D. sechellia* [22] used introgression mapping, allowing for the contributions of individual genomic regions to be assessed independently. In Masly et al. [22], small homozygous pieces of the* D. mauritiana* genome are present in an otherwise homozygous* D. sechellia* genetic background. They found two genomic regions that caused the size of the lobes to shift closer to that of* D. mauritiana*, located near the left telomere and the centromere of the third chromosome. They found four other regions that affected lobe shape, including one near the right telomere of the third chromosome. Regions influencing lobe shape did not overlap those found for lobe size. This demonstrated that individual genomic regions could influence genital morphology and that there is a differential genetic basis for the size and shape aspects of genital morphology in this species pair.

Here, we examine whether the ability of individual genomic regions to influence lobe morphology and the genetic uncoupling of size and shape is present in other species pairs or if it is unique to the* D. mauritiana*-*D. sechellia* species pair. We performed this study in the overlapping species pair of* D. simulans-D. mauritiana*, allowing us to additionally examine whether the same loci would underlie genital morphology differences in related sibling species or if each species in this subgroup owes its unique morphology to separate loci. The sister species* D. simulans* and* D. mauritiana* have been a well-studied example of genital morphology divergence within this subgroup [5, 18, 19, 23]:* D. simulans* males have helmet-shaped lobes, while* D. mauritiana* males have stick-like protrusions [5, 22, 24]. When these two species are crossed, F_1_ hybrid males have an intermediate posterior lobe morphology when compared to the two parental species, while males resulting from a backcross to either parent species produce a continuous range of lobe phenotypes [5, 23], indicating a polygenic nature for lobe morphology, which has been confirmed by QTL mapping [23]. Although genomic regions were located using QTL mapping, it is unknown whether they individually will have an effect on male genital morphology. Indeed, since none of the individual regions had a large effect on the phenotype, it is possible that the effect of a single locus might be undetectable when it is measured individually.

We utilize introgression lines to assess the contributions of individual genomic regions to the divergent genital lobe size and shape between* D. simulans* and* D. mauritiana*. We focused on the three regions identified in Masly et al. [22] on the third chromosome as individually influencing* D. mauritiana* and* D. sechellia *lobe morphology: left telomere, centromere, and right telomere. Previous QTL mapping studies identified these same three regions as contributors to lobe morphology in the* D. simulans-D. mauritiana* species pair [5, 23]. Since the previous work on genital morphology [22] found that some regions of the genome affected the lobes in a direction opposite to expectation (increasing size when they should have decreased size), presumably due to transgressive segregation arising from either additive effects or epistatic interactions with the genetic background, we assess introgressions in both directions: lines that are entirely* D. mauritiana* except for an introgressed* D. simulans* genomic segment (M_S_) and the reciprocal lines that are entirely* D. simulans* except for a* D. mauritiana* introgression (S_M_). We compared the lobe size and shape of these lines to the lobes of the species contributing the majority of the genomic complement and evaluated whether the introgressed genome affected lobe size and/or shape.

## 2. Materials and Methods

### 2.1. Drosophila Stocks

Introgression lines for the third chromosome were previously created [25] by repeated backcrossing of F_1_ hybrids to their parent species and then by several generations of brother-sister mating, paired with molecular genotyping at every generation, to make the introgressions homozygous. Genetic markers were then used to determine the location of the genomic region of the opposite species. The resulting introgression lines of* D. simulans* and* D. mauritiana* contain known inserted regions of the opposite species within their respective genomes (Table 1).

Introgression lines containing each of the three cytological locations important for posterior lobe morphology (left telomere, centromere, and right telomere) were used for dissections of the posterior lobe; we assayed the lines containing the largest introgressions in these regions to increase the likelihood of capturing genetic factors for genital morphology (Table 1) [25]. We have maintained the nomenclature used in McNiven and Moehring [25] for consistency. The three backcrossed* D. mauritiana* lines with known introgressed* D. simulans* genomic regions (M_S_) were line M_S(62)_3 (containing the left telomeric region from* D. simulans*, near cytological band 62), line M_S(82)_6 (containing the centromeric region from* D. simulans*, near cytological band 82), and line M_S(98)_1 (containing the right telomeric region from* D. simulans*, near cytological band 98). The five backcrossed* D. simulans* lines with known* D. mauritiana* genomic regions dissected were lines S_M(62)_1 and S_M(62)_2 (containing the* D. mauritiana* left telomeric region), line S_M(82)_4 (containing the* D. mauritiana* centromeric region), and lines S_M(98)_1 and S_M(98)_5 (containing the* D. mauritiana* right telomeric region).

### 2.2. Comparing Posterior Lobe Area, Length, and Width

**Figure 1:**
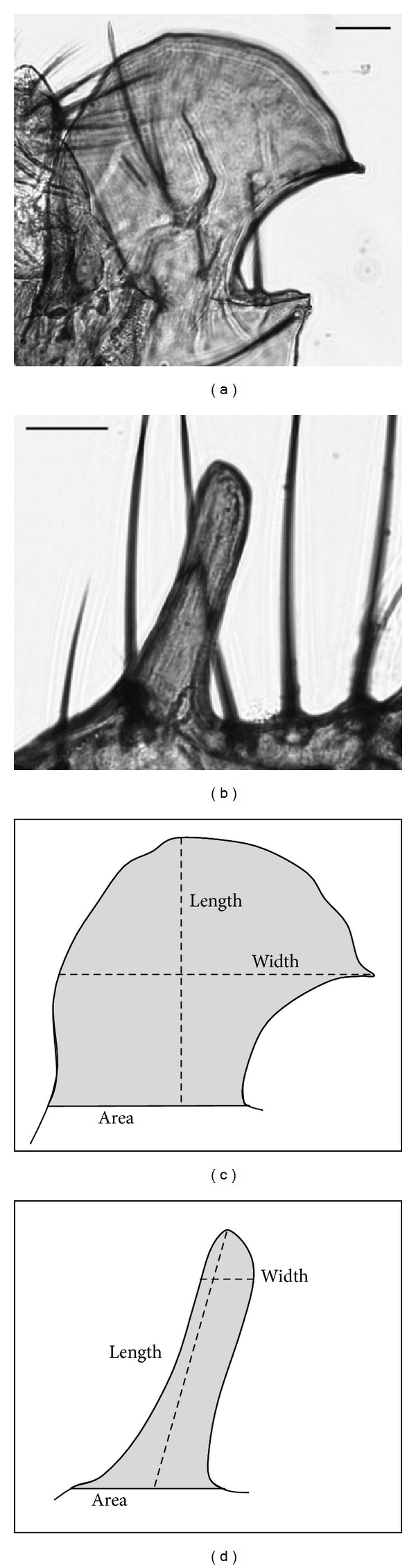
The male posterior lobe in* D. simulans* (a) and* D. mauritiana* (b). A horizontal line was drawn at the base of the arch of* D. simulans* (c) and* D. mauritiana* (d); the area enclosed within the resulting outline was measured as the area; the length from the line to the furthest point was the length; the widest point that was at least 25 *μ*m from the base was the width. Scale bars are 25 *μ*m.

A microknife was used to remove one randomly chosen posterior lobe from the abdomen in TE buffer. A coverslip was then used to ensure that the posterior lobe was observed in a single focal plane. An E100 Nikon compound microscope equipped with a 5-megapixel camera was used to visualize the posterior lobes. All lobe measurements were performed using the computer software NIS-Elements 3.1 (sample size *N* = 10). Lengths were measured in* D. simulans* as the distance from the base of the lobe to the furthest vertical point, as drawn by a line perpendicular to the base; in* D. mauritiana*, the length was measured from the midpoint of the baseline to the furthest point (Figure 1). We found that these two different measures of length in the two species were necessary in order to obtain consistent results due to the general lack of morphological landmarks on the lobes. Widths were measured along a horizontal line at the widest point of the lobe; area was measured by outlining the perimeter of the lobe (Figure 1). All values were first corrected for body size using the tibia length measurements prior to statistical comparison. A one-way ANOVA was used to determine if there was a significant difference in the area, length, or width of the posterior lobes, when comparing the introgressed lines to the parental species comprising the genetic background.

### 2.3. Elliptical Fourier Analysis and Principal Component Analysis

Due to the paucity of morphometric landmarks, an elliptical Fourier analysis was used to represent each posterior lobe's shape (*N* = 10) [5]. We were able to accomplish this because of the 2D nature of the posterior lobe. To do this, the SHAPE program [26] was used to first normalize the posterior lobe shape of males from introgression lines by the area of the lobe in order to correct for size differences and assign a chaincode value. Chaincode is a coding system within the SHAPE software for representing geometrical shapes as numbers. These values were then used to calculate the elliptical Fourier descriptors (EFD) and to visualize them for comparisons. We obtained 20 Fourier harmonics per posterior lobe, which allowed for precise outlines.

To determine how many variables could be used to explain the variation between the introgression lines compared to the wild type lines, a principal components analysis (PCA) [5] was performed, also using the SHAPE program, for each backcross type. The PCA performed using PrinComp, a component of the SHAPE program, is based on the variance-covariance matrix. In both the* D. simulans* and* D. mauritiana*, PC1–PC7 explained at least 90% of the variation observed when comparing introgression lines to the pure-species lines. PC1 and PC2 were evaluated separately using a single-factor ANOVA for differences between the introgression line's genital lobe shape and the lobe shape of the parental species that contributed the genetic background.

## 3. Results

### 3.1. Comparison of Posterior Lobes due to* D. mauritiana* Introgressions

**Figure 2:**
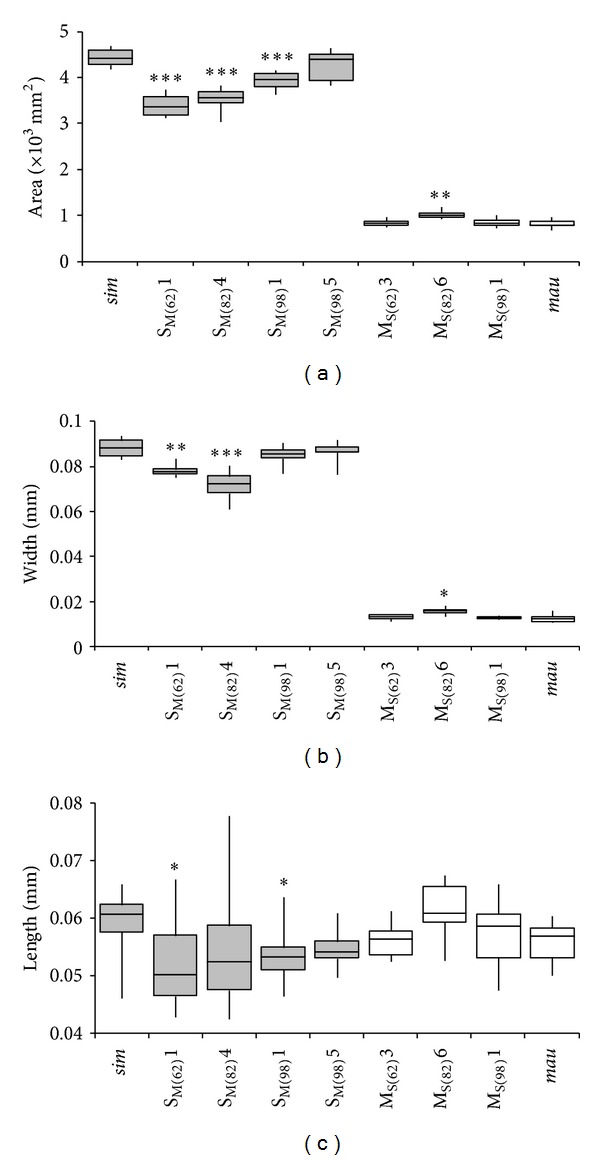
Comparison of posterior lobes in introgression lines versus parental lines. Box plots show the distribution of the uncorrected values for area (a), width (b), and length (c) of the posterior lobes in* D. simulans *FC (*sim*; grey boxes), introgressions of* D. mauritiana* into a* D. simulans* genetic background (S_M_; grey), introgressions of* D. simulans* into a* D. mauritiana* genetic background (M_S_; white), and* D. mauritiana* SYN (*mau*; white). Boxes represent the interquartile range, with the inner horizontal line at the median and the vertical lines denoting the maximum and minimum values (*N* = 10). The introgression lines that were significantly different from the parental line constituting the genetic background, after correction for body size, are marked with *P ≤ 0.05, **P ≤ 0.005, and ***P ≤ 0.0001. Note that, to allow for comparison to Masly et al. [22], the box plot values in the figure are uncorrected for body size; statistical significance, however, was calculated on values that were corrected for body size.

When comparing the overall morphology of the posterior lobes of the introgression lines, the morphology appeared to be species-specific and predominantly in accordance with the backcross genetic background (Table 2; Figure 2). Lobe area showed a strong correlation with both lobe width (values from 0.64 to 0.93) and lobe length (values from 0.50 to 0.80), with a stronger correlation for lobe width in all lines. Posterior lobes in the parental* D. simulans* males were significantly wider and longer and had a greater mean area when compared to the posterior lobes of males containing the* D. mauritiana *introgression in line S_M(62)_1 (df = 18; *P* = 0.001, *P* = 0.032, and *P* < 0.0001, resp.). The introgression line from the same region, S_M(62)_2, also displayed reduced lobe size [lobe area (mm^2^)/tibia size (mm) = 8.77 ± 0.53 (×10^3^)], but the lobes appeared to be aberrant and malformed in some of the dissections performed (2/10). These sporadic differences observed in the one line are unlikely to be due to the species-specific introgression as they were not observed in the overlapping line S_M(62)_1. As such, we removed this line from further analyses. However, if the observed differences were due to the introgression, then the loci for shape would fall within the small region of unknown genotype on the border between the markers assessed as being the introgression versus parental genotype.

Significantly greater width and area were also observed for the introgression line S_M(82)_4 (df = 18; *P* < 0.0001, *P* < 0.0001, resp.). The length and area of the posterior lobe were also significantly different when comparing the posterior lobes of parental* D. simulans* males to those from the introgression line S_M(98)_1 (df = 18, *P* = 0.020, *P* < 0.0001) and approached significance for width (df = 18, *P* = 0.063). The posterior lobes from the partially overlapping introgression line S_M(98)_5 did not differ significantly in mean width, length, or area when compared to the posterior lobes of parental* D. simulans*. It should be noted that, for practical reasons, we used a slightly different protocol for measuring length in* D. simulans* males than in* D. mauritiana* males, and this may have biased our results for this phenotype. However, since the lobes of introgression males largely resembled those of the parental species comprising the genetic background, these different measures likely had a minor, if any, effect on our assessment of length in introgression males. None of the introgression lines had a significant difference in tibia length compared to pure-species* D. mauritiana*. There was a slightly negative, and nonsignificant, correlation between individual measures of lobe area and tibia length (*r* = −0.016, *P* = 0.92).

**Figure 3:**
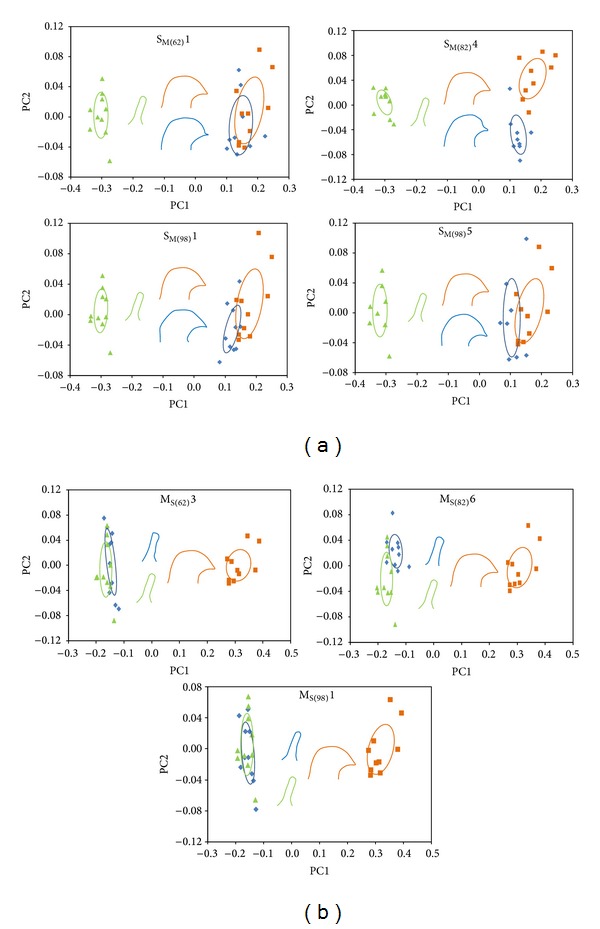
Shape measurements in introgression lines. The distribution of the first two principal components obtained from an elliptical Fourier analysis for pure-species* D. mauritiana* (green triangles) and* D. simulans* (orange squares) compared to introgression lines (blue diamonds) containing (a) introgressed regions of* D. mauritiana* in an otherwise* D. simulans* genetic background (S_M_) or (b) introgressed regions of* D. simulans* in an otherwise* D. mauritiana* genetic background (M_S_). Ellipses represent the standard deviation centered on the mean value for each group. A representative lobe shape for each line is shown in the same color as the group it represents.

In the principal component analysis, PC1-9 accounted for 95.0% of the variance in the S_M_ lines, with the majority of the variance explained by PC1 (35.0%) and PC2 (21.4%). PC1, as expected, largely indicated differences in lobe area. Comparisons of PC1 and PC2 between the introgression lines and the parental* D. simulans* line identified which introgressed regions affected the species-specific shape of the posterior lobe (Figure 3(a)). The shape of the posterior lobe was not significantly affected by an introgressed region near the left telomere in line S_M(62)_1. However, the introgressed region at the centromere (in line S_M(82)_4) significantly affected both PC1 (df = 1, *F* = 9.71, *P* = 0.006) and PC2 (df = 1, *F* = 38.41, *P* < 0.0001), while both lines containing an introgressed segment at the right telomere (S_M(98)_1 and S_M(98)_5) had a significant difference in PC1 (df = 1, *F* = 15.27, *P* = 0.001; df = 1, *F* = 9.58, *P* = 0.007, resp.) but not in PC2.

### 3.2. Comparison of Posterior Lobes due to* D. simulans* Introgressions

As with the above introgressions, the overall morphology of the posterior lobes in lines with an introgression from* D. simulans* was species-specific and predominantly similar to that of the parental genetic background,* D. mauritiana*. In contrast to what was seen for S_M_ lines, the M_S_ lines showed generally weaker and more variable correlations between lobe area and lobe width or length: M_S(62)_3 (width: 0.72, length: 0.65), M_S(82)_6 (width: 0.31, length: 0.28), and M_S(98)_1 (width: 0.44, length: 0.51). There was a significant difference in the mean width and area of the posterior lobe when comparing the parental* D. mauritiana* to the introgression line M_S(82)_6 (df = 18; *P* = 0.043, *P* = 0.003, resp.). The introgression lines from the other two cytological locations, M_S(62)_3 and M_S(98)_1, did not show any statistically significant difference in mean width, length, or area of the posterior lobe when compared to those of the parental* D. mauritiana* males, but M_S(62)_3 did approach significance for width (df = 18, *P* = 0.059). As with the* D. mauritiana* introgression males, our different protocol for length measurements in the two parental species may have biased our results, but this is unlikely. None of the introgression lines had a significant difference in tibia length compared to pure-species* D. simulans*, and there was a nonsignificant negative correlation between lobe area and tibia length (*r* = −0.12, *P* = 0.40).

In the principal component analysis, PC1-9 accounted for 95.6% of the variance in the M_S_ lines, with the majority of the variance explained by PC1 (41.0%) and PC2 (24.8%). As with the S_M_ introgressions, PC1 for the M_S_ lines largely indicated differences in lobe area. Comparisons of PC1 and PC2 between the introgression lines and the parental* D. mauritiana* lines found that regions at the left telomere and centromere affected the species-specific shape of the posterior lobe (Figure 3(b)). Line M_S(62)_3, which has an introgression at the left telomere, significantly differed in shape for PC1 (df = 1, *F* = 4.95, *P* = 0.039) but not for PC2. Line M_S(82)_6, with an introgression at the centromere, was significantly different in shape for both PC1 (df = 1, *F* = 10.63, *P* = 0.004) and PC2 (df = 1, *F* = 7.77, *P* = 0.012), while line M_S(98)_1, with an introgression at the right telomere, did not significantly differ in either aspect of shape.

## 4. Discussion

**Figure 4:**
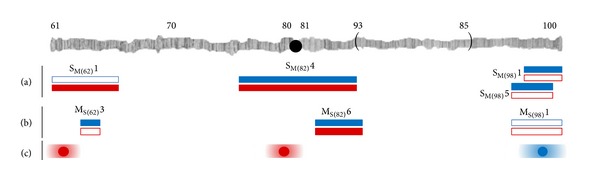
Genomic regions that significantly affect species-specific lobe size and shape. The third chromosome is shown at the top of the figure: the black circle is the centromere, and the brackets represent a fixed inversion difference between* D. melanogaster* compared to* D. simulans*,* D. mauritiana*, and* D. sechellia*. Numbers above the chromosome are approximate cytological regions. (a) Introgressions of* D. mauritiana* into an otherwise* D. simulans* genetic background (S_M_) and (b) introgressions of* D. simulans* into an otherwise* D. mauritiana *genetic background (M_S_) that significantly affected the area (filled red bars; data from Table 2, Figure 2) or shape (filled blue bars; data from Figure 3) of the posterior lobe; open bars indicate nonsignificance for these traits. The length of the bars approximates the size of the introgressed segment. (c) Introgressions of* D. mauritiana* into an otherwise* D. sechellia* genetic background (adapted from [22]) that significantly affected the area (red circles) or shape (blue circles) of the posterior lobe. The circles represent the midpoint of the introgression; there is a fade-out on the periphery to indicate that the boundaries of these introgressions are not published. All of the introgressions in (a), (b), and (c) are aligned with their representative cytological positions on the chromosome.

The* Drosophila melanogaster* subgroup is highly divergent with regard to the shape of the male posterior lobe. Aside from the posterior lobe, there are no other significant differences in overall body morphology between the species, and there is very little correlation between body size and lobe size or shape [5, 22, 27, 28], although lobe size was found to be correlated with tibia length in a recent study [29]. The differences in genital morphology are caused by multiple genomic regions that, in general, act additively to contribute to the shape and size of the posterior lobe [5, 22, 23, 28, 30]. A previous study that utilized quantitative trait locus (QTL) mapping found that the genetic regions that determine size and shape differences between the posterior lobes of* D. simulans* and* D. mauritiana* were indistinguishable [5], and therefore lobe, size, and shape were considered genetically linked in these species. In contrast, a study utilizing introgression lines found that the genomic regions influencing the species-specific difference in size for the lobes of* D. mauritiana* compared to* D. sechellia* were often different from those that conferred differences in lobe shape [22], indicating that differences in lobe size and shape in these species have separate genetic bases. Our findings agree with both of these previous studies: some regions of the genome contribute to both size and shape, while others affect either size or shape (Figures 4(a) and 4(b)). Thus, there is genetic linkage (association, physical linkage, or pleiotropy) between some loci influencing size and shape differences between* D. simulans* and* D. mauritiana*, while other loci either are not linked or are linked with loci whose effect is too small to be detected in this study.

When portions of the* D. mauritiana* genome were introgressed into* D. sechellia*, introgressions at the left telomere and centromere influenced size, while an introgression at the right telomere altered the shape of the* D. sechellia* lobe towards a* D. mauritiana*-like appearance (Figure 4(c)) [22]. We found that these three regions of* D. mauritiana *have the same effect on* D. simulans* lobe morphology as the one they have on* D. sechellia* lobe morphology, with an additional effect on shape for the centromeric region; this additional effect is likely due to the large size of this introgression (Figures 4(a) and 4(c)), as the significant introgression into* D. sechellia* does not span the entire length of the genomic region we introgressed into* D. simulans* ([22]; J. P. Masly, personal communication). One of the three regions (at the centromere) was also implicated as contributing to intraspecific variation in lobe morphology within* D. melanogaster* [30]. It is therefore possible that there may be a similar genetic underpinning for genital divergence in this species group; this is not surprising, as it makes sense that the same developmental pathways could be influenced by selection during these species' divergence.

Only one of the three regions had the same effect on lobe morphology when they were introgressed in the alternate direction; that is,* D. simulans *genome introgressed into* D. mauritiana* (Figure 4(b)). For example, the introgression at the left telomere affects lobe shape rather than size, demonstrating that the genes in this region likely do not have the same effect on the two species as the portion of the genome that is introgressed in M_S(62)_3 is also present in line S_M(62)_1. Likewise, the introgression M_S(98)_1, which does not have an effect on lobe morphology, contains all of the equivalent genomic regions present in the significant lines SM(98)1 and S_M(98)_5 and contains all of the regions present in the* D. mauritiana* introgression that significantly affected lobe shape in* D. sechellia* ([22]; J. P. Masly, personal communication). Thus, there is divergence in how individual genomic regions influence morphology, and the loci within these regions appear to interact with their genetic background. Two of these regions, although they had a significant effect on shape (M_S(62)_3) or area and shape (M_S(82)_6) in our study, do not directly overlap the location of the introgressions of* D. mauritiana* into* D. sechellia* that were shown to have a significant effect on lobe area ([22]; J. P. Masly, personal communication). Thus, it appears that there may be regions of the genome that harbor multiple loci that have the potential of contributing to the variation in lobe morphology in this species group.

Although they were not identified as significantly influencing lobe area in the* D. mauritiana*-*D. sechellia* species pair, these regions were found to influence lobe phenotype, but in an unexpected direction [22]. The introgression* 2H3(B)* overlaps the same region covered here by M_S(62)_3, but in the former study the introgressed piece of* D. mauritiana* caused the lobe to have a larger size than either parental species and skewed the shape away from that of* D. mauritiana*. Likewise, introgression* 2K3(A)* overlaps M_S(82)_6 but increased the size above that of either parental species. This skew in lobe phenotype away from the expected direction (i.e., the phenotype became even more dissimilar from* D. mauritiana*) was most likely due to epistatic interactions or transgressive segregation [22]. In contrast, none of our introgressions produced a significant phenotype in the opposite direction to the expected in either size (Table 1, Figure 2) or shape (Figure 3). Thus, it appears that the observed skew due to introgressions of* D. mauritiana* for these regions was due to their placement into a* D. sechellia *genetic background; when they are placed into a* D. simulans* genetic background, they significantly affect size and/or shape in the expected direction.

As was found in the* D. mauritiana*-*D. sechellia *species pair [22], our results also indicate that single genomic regions can significantly modify genital morphology, suggesting that individual genes may have a strong enough effect on lobe morphology that it may be possible to map their separate locations. This result is still somewhat unexpected as lobe morphology in the* D. simulans*-*D. mauritiana* species pair was previously mapped to more than 19 genomic regions [23], making future fine-mapping appear impossible as each region was assumed to have too small of an effect to be individually detectable by reasonable means. While this still may be the case, as our introgressed regions are large and may harbor multiple loci of small effect, the lobe area shifted by 22–24% in our significant lines, making the phenotype relatively pronounced, enhancing the prospect of future fine-mapping.

The region at the right telomere that was significant for both* D. simulans*-*D. mauritiana* and* D. mauritiana*-*D. sechellia* lobe shape harbors a candidate gene for posterior lobe morphology [22, 31]. The* D. melanogaster* gene known as* Drop* (*Dr*), at cytological location 99B, has been identified as important in sex determination.* Dr* is repressed in females during development and, when mutated in* D. melanogaster* males, leads to misshapen posterior lobes [31]. A comparison of published sequences [32] confirmed that there is a homolog for* Dr* in both* D. simulans* and* D. mauritiana* in the same cytological region, making this gene a strong candidate for variation in lobe morphology in this species pair.

The posterior lobes are thought to play a role in both copulation and fertilization [19–21], and as such, divergence in lobe morphology could influence male mating success with females of another species. The same telomeric and centromeric regions on the 3rd chromosome that affect genital shape morphology here have also been found to affect mating behavior in* D. simulans*-*D. mauritiana* [25, 33] and* D. simulans*-*D. melanogaster* [34]. We can examine whether the different lobe morphology induced by the introgressions has an impact on mating behavior by testing the behavior of the introgression lines. A previous study examined three of the same introgression lines used here for their effect on male mating success [25]. When* D. mauritiana* males harboring a* D. simulans *introgression were paired with* D. mauritiana* females, the males with an introgression at the centromere (M_S(82)_6) and right telomere (M_S(98)_1) had a significant reduction in copulation success, while males with an introgression at the left telomere (M_S(62)_3) did not have reduced mating success. As these results do not align with our significant results for alteration in lobe size or shape (Figure 4(b)), differences in male mating success do not appear to be induced by the variation in lobe morphology observed for these lines, but additional tests are required to rule out linkage between these traits. Additionally, genes for a sexually selected trait are again found to localize near the centromere and telomeres, a trend that is potentially widespread [34].

## Figures and Tables

**Table 1:** Location of introgressions.

Line name^1^	Introgressed region: base positions^2^	Introgressed region: cytological^3^
S_M(62)_1	(3L) 41-8706	61A-67B
S_M(62)_2	(3L) 41-8700	61A-67B
S_M(82)_4	(3L) 16451-(3R)4871	74A-92F
S_M(98)_1	(3R) 23001-telomere	98A-telomere
S_M(98)_5	(3R) 21267-26170	96E-100A
M_S(62)_3	(3L) 1457-3921	62B-64B
M_S(82)_6	(3L) 22342-(3R)5411	80F-92D
M_S(98)_1	(3R) 21267-telomere	96E-telomere

^1^The lines are either a piece of *D. mauritiana* genome in an otherwise *D. simulans* genetic background (S_M_) or a piece of *D. simulans* genome in an otherwise *D. mauritiana *genetic background (M_S_) for three segments of the third chromosome (cytological region 62, 82, or 98). The line number is consistent with the designation previously used for the same lines [25].

^2^The base positions are in kilobases, numbered from the telomere for the left arm (3L) and from the centromere for the right arm (3R) of the third chromosome. The region that is listed spans from the markers that had the genotype of the genomic background, encompassing the markers that had the introgressed parent's genotype; thus, the size of the actual region is likely smaller than the listed region.

^3^The cytological position is that of the homologous region in *D. melanogaster*.

**Table 2:** Tibia length, posterior lobe area, lobe length, and lobe width measurements.

Genotype	Tibia length (mm)	Lobe area (×10^3^ mm^2^)	Lobe width (mm)	Lobe length (mm)
*D. simulans* FC	0.3743 ± 0.0167	4.432 ± 0.112	0.0882 ± 0.0024	0.0593 ± 0.0035
S_M(62)_1	0.3800 ± 0.0235	3.401 ± 0.137∗∗∗	0.0769 ± 0.0025∗∗	0.0517 ± 0.0045∗
S_M(82)_4	0.3842 ± 0.0132	3.534 ± 0.162∗∗∗	0.0731 ± 0.0042∗∗∗	0.0552 ± 0.0060
S_M(98)_1	0.3837 ± 0.0100	3.942 ± 0.112∗∗∗	0.0860 ± 0.0025	0.0534 ± 0.0033∗
S_M(98)_5	0.3568 ± 0.0165	4.266 ± 0.207	0.0861 ± 0.0027	0.0546 ± 0.0020
*D. mauritiana* SYN	0.3742 ± 0.0123	0.832 ± 0.053	0.0127 ± 0.0010	0.0566 ± 0.0022
M_S(62)_3	0.3551 ± 0.0151	0.847 ± 0.041	0.0135 ± 0.0007	0.0564 ± 0.0017
M_S(82)_6	0.3915 ± 0.0081	1.033 ± 0.053∗∗	0.0152 ± 0.0012∗	0.0630 ± 0.0035
M_S(98)_1	0.3760 ± 0.0080	0.743 ± 0.050	0.0130 ± 0.0003	0.0582 ± 0.0036

Comparison to *D. simulans* FC (for S_M_) or *D. mauritiana* SYN (for M_S_): *P ≤ 0.05, **P ≤ 0.005, and ***P ≤ 0.0001. All values were adjusted for body size by dividing by tibia length prior to statistical analysis.
